# Complete Genome Sequence of Pittsburgh Sewage-Associated Virus 1

**DOI:** 10.1128/genomeA.01460-17

**Published:** 2018-03-15

**Authors:** Paul G. Cantalupo, James M. Pipas

**Affiliations:** a Department of Biological Sciences, University of Pittsburgh, Pittsburgh, Pennsylvania, USA

## Abstract

We present the complete genome sequence of a virus found in raw sewage collected in Pittsburgh, PA, USA. Pittsburgh sewage-associated virus 1 (PSAV1) encodes one large open reading frame with conserved domains typically found in the *Picornavirales* order of viruses. PSAV1 is closely related to *Biomphalaria* virus 2 (BV2).

## GENOME ANNOUNCEMENT

The *Picornavirales* order contains several virus families, such as *Picornaviridae*, *Dicistroviridae*, and *Secoviridae*, that infect mammals, insects, and plants. These viruses are small and nonenveloped and contain a monomeric single-strand positive-sense RNA genome of 7 to 10 kb in length. Usually they contain one or two large open reading frames (ORFs), which generate a polyprotein that is subsequently cleaved by a viral protease into functional polypeptides. The global diversity of these and other RNA viruses continues to be revealed through deep sequencing studies (1, 2). Raw sewage is a rich biomatrix that supports both multicellular and microorganism populations and has been shown to contain an abundance of novel viruses (3–5).

Viral nucleic acid from Pittsburgh raw sewage was extracted and sequenced (3). After nontemplate sequences were removed from the raw reads (NCBI Sequence Read Archive accession number SRR315458), reads were assembled with Celera assembler (v. 7.0) using a 3% utgErrorRate setting (6). The longest contig generated, 8,745 bp, was annotated in 2012 by a BLASTX (7) search, which showed it to be distantly related to *Dicistroviridae*. The termini of the genome were completed with 5′ and 3′ rapid amplification of cDNA ends (RACE), which added 49 and 201 bp to the 5′ and 3′ ends, respectively. The 3′ RACE revealed that the genome is poly(A) tailed. The 8,995-bp genome sequence was confirmed by Sanger sequencing of staggered PCR amplicons across the genome.

A BLASTN search showed 78% identity between PSAV1 and *Biomphalaria* virus 2 (BV2) (GenBank accession number KY024322), an unclassified *Picornavirales* virus (8). PSAV1 contains one ORF that is predicted to encode a 2,668-amino acid (aa) polyprotein which is 86% identical to the BV2 polyprotein. PSAV1 polyprotein contains several functional domains, as identified by using CD-Search (9), including RNA helicase, protease, RNA-dependent RNA polymerase (RdRp), and three capsid domains. Since the proteolytic cleavage sites are unknown, the putative start and end coordinates of each polypeptide were based on the CD-Search results. The RNA-helicase (107 aa, 2,502 to 2822 nucleotides [nt]) contains a GX_4_GKS motif and a KX_5_SX_5_S/TNN motif, which have been shown to be responsible for deoxynucleoside triphosphate (dNTP) binding and ATP hydrolysis (10). PSAV1 protease (196 aa, 3,558 to 4,145 nt) was putatively identified as a cysteine protease due to a cysteine at residue 1079 replacing the serine residue found in many RNA virus proteases (11). The RdRp (474 aa, 4,248 to 5,669 nt) polypeptide contains motifs A (DX_4_D), B (PXG), and C (GDD), which are characteristic of a picornavirus RdRp (12). We were not able to identify a VPg protein in PSAV1. The three viral capsid polypeptides are predicted as capsid1 (195 aa, 6,318 to 6,902 nt), capsid2 (185 aa, 7,299 to 7,853 nt), and capsid3 (249 aa, 7.959 to 8,705 nt).

The phylogenetics of PSAV1 show that it is closely related to BV2, a snail virus. Since PSAV1 was isolated from raw sewage, its host is unknown. Both viruses share no significant homology to other viruses. These findings suggest that more members of this distant *Picornavirales* clade remain to be discovered.

### Accession number(s).

The genome sequence of PSAV1 has been deposited in GenBank under the accession number MG552116.
